# Effect of abdominal drawing-in maneuver during hip extension on the muscle onset time of gluteus maximus, hamstring, and lumbar erector spinae in subjects with hyperlordotic lumbar angle

**DOI:** 10.1186/1880-6805-33-34

**Published:** 2014-11-27

**Authors:** Taewoo Kim, Youngkeun Woo, Yongwook Kim

**Affiliations:** Department of Rehabilitation Science, The Graduate School, Jeonju University, 303, Cheonjam-ro, Wansan-gu, Jeonju, Jeonbuk-do, 560-759 Korea Republic; Department of Physical Therapy, College of Medical Sciences, Jeonju University, 303 Cheonjam-ro, Wansan-gu, Jeonju, Jeonbuk-do, 560-759 Korea Republic

**Keywords:** Abdominal drawing-in maneuver, Gluteus maximus, Muscle onset time, Lumbar lordosis

## Abstract

**Background:**

The abdominal drawing-in maneuver (ADIM) is used to prevent abnormal movements of the lumbar spine and pelvis during therapeutic exercises. This study compared the effects of ADIM on the muscle onset time of the hamstring, gluteus maximus, and erector spinae muscles during prone hip extension exercise in subjects with or without hyperlordotic lumbar angle. Forty healthy adults (18 male, 22 female) were recruited for this study.

**Methods:**

The lumbar lordotic angles and pelvic tilt angles of the subjects were measured using the Avaliação postural analysis software. The subjects were divided into two groups: the lumbar hyperlordotic angle (LHLA) and lumbar normal lordotic angle (LNLA) groups. The muscle contraction onset time of the hamstring, gluteus maximus, and erector spinae were assessed using surface electromyography.

**Results:**

During ADIM application, the muscle contraction onset time of the gluteus maximus was significantly increased in the LHLA group compared with the LNLA group.

**Conclusions:**

ADIM application during prone hip extension was more effective for gluteus maximus onset time in the LHLA group. Therefore, ADIM during prone hip extension may be useful for gluteus maximus training in individuals with lumbar hyperlordosis.

## Background

Abnormal alignment and muscle imbalance of the lumbar spine causes stress to the surrounding structures, which can lead to musculoskeletal dysfunctions such as lumbar hyperlordosis and excessive pelvic anterior tilting [1–3]. Additionally, such abnormal alignment and muscle imbalance of the lumbar spine and pelvic regions can limit the mobility of the lumbar spine and weaken the hip extensors [4, 5]. Therefore, correct alignment of the lumbar and pelvis is required and depends upon the flexibility of the hip joint and spine, in addition to maintenance of the correct lumbar lordotic angle [6]. In the clinical setting, the abdominal drawing-in maneuver (ADIM) is used to prevent abnormal movements of the lumbar region and pelvis during hip extension [7]. Furthermore, ADIM is known to guide proper alignment of the lumbar spine and pelvis during the prone hip extension exercise, which represents a general clinical method of strengthening weakened hip extensors [8].

Sakamoto et al. [9] reported the occurrence of a consistent sequence of muscle contractions of the hamstring (HAM), gluteus maximus (GM), and lumbar elector spinae (LES) during hip extension, and suggested that the sequential muscle activation at the correct time was critical for the effective movement of the lumbar region and pelvis. However, the onset times of muscle contraction can be altered by an abnormal lumbar lordotic angle and excessive pelvic anterior tilting, which in turn delays the response time of the abdominal muscles and GM in daily activity [10]. Nygren Pierce and Lee [11] reported that a delay in GM muscle activity was caused by pelvic instability and changes in surrounding muscle lengths during hip extension. In this way, the altered onset times of trunk and hip extension could decrease the stability of the lumbar spine and pelvis during walking, degrade the mechanical efficiency of the pelvic and lumbar motion, and cause low back pain [12].

Previous studies reported that application of ADIM stabilized the lumbar spine and pelvis during hip extension, and was effective for pain relief and re-education of muscle functions [7, 13]. However, previous studies investigating the effects of ADIM during hip extension on the muscle activity of the HAM, GM, and LES were performed in patients with low back pain, and did not consider the lumbar hyperlordotic angle in the subjects. Therefore, the purpose of this study was to investigate the effects of ADIM on the muscle onset time in the HAM, GM, and LES during hip extension in subjects with hyperlordotic angle but with no lower back pain. In addition, we evaluated the clinical utility of ADIM during hip extension in subjects with lumbar hyperlordotic angle. We hypothesized that the application of ADIM in subjects with lumbar hyperlordotic angle affects the onset time of the HAM, GM, and LES during prone hip extension, and that a significant difference exists between the lumbar hyperlordotic angle (LHLA) group and the lumbar normal lordotic angle (LNLA) group.

## Methods

### Participants

The subjects of this study were 40 adults (male, n = 18; female, n = 22) who understood the objective of this study and consented to participate in this study. Written informed consent was obtained from the patient for the publication of this report and any accompanying images. The study complied with the ethical standards of the Declaration of Helsinki and the Jeonju University Research Ethics Committee approved the experimental protocol. An inter-group comparison experiment design was used, where subjects were divided into LHLA and LNLA groups. A paired matching design was used, so that subjects in the LNLA group demonstrated similar general and medical characteristics including height, weight, and sex to their counterparts in the LHLA group. Before the experiment began, the lumbar lordotic angle and the pelvic tilt angle were measured. Those whose lumbar lordotic angle was 45° or below and pelvic anterior tilt angle was 15° or above were assigned to the LHLA group; the remaining subjects were assigned to the LNLA group.

The following subjects were included: i) subjects without musculoskeletal disabilities such as a lower back pain and contracture of lower extremity, ii) subjects without neurological disorder, and iii) subjects without pain in the lumbar region and hip during hip extension. The general characteristics of the subjects are shown in Table 1. There were no significant differences in age, sex, height, and weight between the two groups (*P* >0.05). However, there were significant differences in the average lumbar lordotic angle and pelvic anterior tilt angle between the two groups (*P* <0.05).

**Table 1:** **Descriptive characteristics of the participants (n = 40)**

	LHLA (n = 20)	LNLA (n = 20)	Significance
	Mean ± SD	Mean ± SD	
Age (years)	22.7 ± 3.8	23.2 ± 3.2	NS
Gender			NS
Male	9 (45%)	9 (45%)	
Female	11 (55%)	11 (55%)	
Height (cm)	165.7 ± 9.1	164.5 ± 10.1	NS
Weight (kg)	60.4 ± 8.3	62.4 ± 6.9	NS
Lumbar lordotic angle (°)	36.2 ± 2.1	60.6 ± 4.6*	*P* <0.05
Pelvic tilt angle (°)	16.9 ± 1.3	7.1 ± 1.9*	*P* <0.05

LHLA, Lumbar hyper lordotic angle; LNLA, Lumbar normal lordotic angle; SD, Standard deviation; *indicates a significant differences between the groups.

### Measurement of lumbar lordotic and pelvic anterior tilt angle

**Figure 1:**
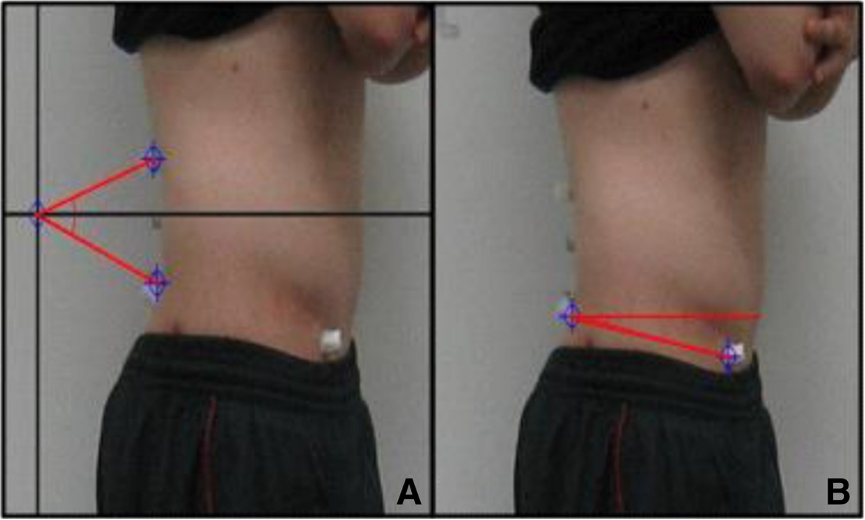
**Experimental measurement of lumbar lordotic (A) and pelvic anterior tilt (B) angles.**

Measurement of lumbar lordosis and pelvic anterior tilt angle was conducted with subjects standing with their feet shoulder-width apart, and with their arms folded and hands on their chest, their sides were then photographed. The experimenter palpated subjects’ T12, L3, and L5 vertebrae, anterior superior iliac spine (ASIS), and posterior superior iliac spine (PSIS), and attached 13-mm marker points prior to taking photographs. Photographs were taken at a distance of 2.4 m from the subject, with the tripod height horizontally aligned with subjects’ pelvises (Figure 1).

To measure the lumbar lordotic angle, the subjects remained in the standing position, next to a plumb line, 15 cm from the wall. The lumbar lordotic angle was defined as the angle between two lines made by T12 and L5 markers at the horizontal L3 level. The pelvic tilting angle was defined as the angle between the horizontal line and the line created by ASIS and PSIS (Figure 1). To maintain positioning, a rectangular-shaped wooden frame (15 × 60 cm) was stationed between the wall and the subject. A wooden bar (7.5 cm in length) was placed between the participant’s feet to ensure correct posture. Photogrammetry was used to assess the lumbar lordotic and pelvic tilting angles. The reliability of lumbar lordosis and pelvic tilt angle measurements is high (*r* = 0.86–0.98) [14].

### Electromyography (EMG) protocol

A Delsys-Tringno EMG was used to collect EMG data, and the EMG signals collected from each muscle were converted to digital signals and processed by Works Acquisition, an EMG analysis software application for PCs. The sampling rate of EMG signals was 2,000 Hz, and the EMG frequency bandwidth was restricted to 20 to 500 Hz. The onset time of each muscle was calculated using the raw signal, following collection of EMG data. The time at which muscle activity deviated by at least two standard deviations from the mean muscle activity was defined as the muscle contraction onset time [15]. Measurements were repeated 10 times, with mean muscle onset times used in the statistical analysis.

### EMG data collection for each muscle

Before measuring EMG signals, any hair on the skin was shaved, which was then cleaned with an alcohol swab before electrodes were attached for EMG measurement. The GM electrode was attached in the center of the line that connects from the lateral angle below the sacral vertebra to the greater trochanter [16]. The HAM electrode was medially located on the biceps femoris muscles 15 cm below the ischial tuberosity [16]. The LES electrode was attached to the muscle belly that is 2 cm away laterally from the spinous process of the first lumbar vertebra [16].

**Figure 2:**
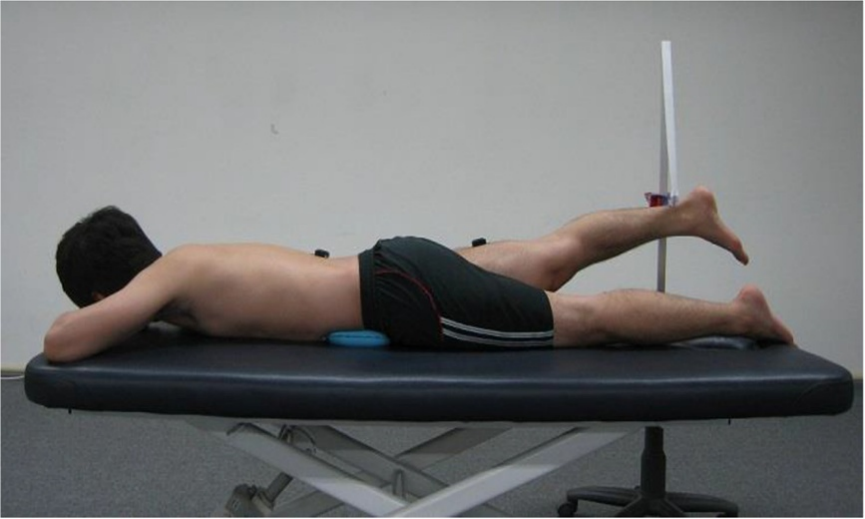
**Experimental posture used to measure hamstring, gluteus maximus, and lumbar erector spinae muscle onset times during prone hip extension.**

Before beginning the experiment, all participants were introduced to the experimental method. The experiment was conducted with subjects in a prone position. A horizontal bar was placed over subjects’ ankle joints, such that they touched the horizontal bar when hip extension reached 10°, measured using a goniometer (Figure 2). To maintain constant movement and synchronize with the EMG measurement during prone hip extension, an electronic metronome was used, which provided initial visual information by displaying each second on the screen. EMG measurement and the electronic metronome were initialized simultaneously. Prior to measurement, subjects were permitted to practice the hip extension motion three times. Knee bend and hip rotation were prohibited during performance of the hip extension motion.

ADIM was performed by both the LHLA and LNLA groups, and was monitored using a pressure biofeedback unit, which was positioned under the lower abdomen of subjects while in the prone position. With the pressure set to 70 mm Hg, subjects commenced ADIM simultaneous with metronome initialization, and lowered the pressure to 60 mmHg, which was then maintained. Subjects were directed to perform the hip extension motion simultaneously with the appearance of the third display on the metronome [13].

### Statistical analysis

For statistical analysis, IBM/SPSS Ver. 20.0 (IBM, Armonk, NY, USA) was used, and the Shapiro-Wilk tests were performed to find the normal distribution of all data. An independent *t*-test was used to verify the differences in general characteristics between the two subject groups. Four separate 2 × 2 analyses of variance determined main and interaction effects for each tested muscle. The within-subject factor was condition (two levels: with and without an ADIM). The between-subject factor was lordotic angle (two levels: LHLA and LNLA). An independent *t*-test was conducted to examine the differences of differential value of each muscle onset time between two groups. A value of *P* <0.05 was taken to indicate statistical significance.

## Results

There was no significant condition × lordotic angle interaction for HAM (F_1,38_ = 0.672, *P* = 0.416) or LES muscle onset time (F_1,38_ = 0.246, *P* = 0.622). However, there was a significant condition × lordotic angle interaction for the muscle onset time of the GM (F_1,38_ = 6.760, *P* = 0.012).

**Figure 3:**
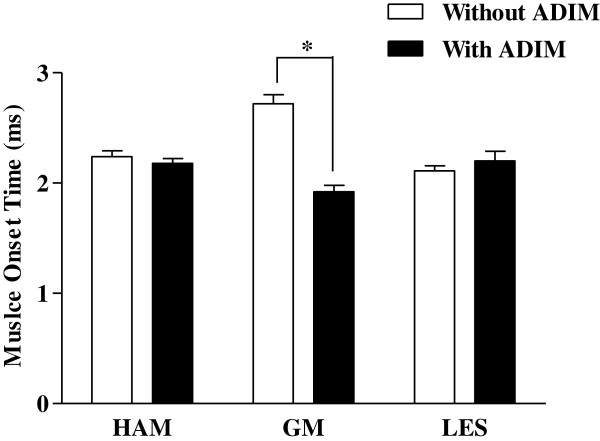
Muscle onset time during prone hip extension with and without performance of the drawing-in maneuver in the lumbar hyper-lordotic angle group (mean ± SD; * ***P*** **<0.01).**

There was no significant main effect for condition for the muscle onset time of the HAM (F_1,38_ = 0.000, *P* = 1.000), GM (F_1,38_ = 1.703, *P* = 0.197), or LES (F_1,38_ = 0.145, *P* = 0.705). An intra-group comparison of the HAM muscle onset time in the LHLA group showed that the muscle onset time was 2.24 ± 0.24 ms without ADIM application and 2.18 ± 0.19 ms with ADIM application, but this change was not significant (*P* >0.05; Figure 3). For the GM, the muscle onset time was 2.72 ± 0.37 ms before ADIM application and 1.92 ± 0.27 ms after ADIM application, and this difference was significant (*P* <0.01; Figure 3). For the LES, the muscle onset time was 2.11 ± 0.21 ms before ADIM application and 2.20 ± 0.40 ms after ADIM application, but this difference was not significant (*P* >0.05; Figure 3). There was no significant main effect of lordotic angle on HAM (F_1,38_ = 0.035, *P* = 0.853), GM (F_1,38_ = 0.348, *P* = 0.558), or LES (F_1,38_ = 0.211, *P* = 0.648) muscle onset time.

An inter-group comparison revealed that the HAM muscle onset time, between with or without ADIM, was 0.07 ± 0.27 ms in the LHLA group and 0.09 ± 0.22 ms in the LNLA group. However, this difference was not significant (*P* >0.05; Table 2). In contrast, a significant difference was observed in the differential value of the GM muscle onset time in the LHLA group (-0.53 ± 0.47 ms) compared with the LNLA group (0.16 ± 0.61 ms) (*P* <0.01). No significant difference was observed between groups in the differential value of LES muscle onset time (*P* >0.05; Table 2).

**Table 2:** **Mean and standard deviations (SD) of differential value of muscle onset time between with and without ADIM in each group (n = 40)**

	LHLA (n = 20)	LNLA (n = 20)	Significance
HAM (ms)	-0.07 ± 0.27	0.09 ± 0.22	NS
GM (ms)	-0.53 ± 0.47	0.16 ± 0.61	*P* <0.01
LES (ms)	0.01 ± 0.38	0.04 ± 0.55	NS

ADIM, Abdominal drawing-in maneuver; LHLA, Lumbar hyper lordotic angle; LNLA, Lumbar normal lordotic angle; HAM, Hamstring; GM, Gluteus maximus; LES, Lumbar erector spinae.

## Discussion

This study examined the effects of AIDM on the muscle onset time of the HAM, GM, and LES, and assessed the clinical applicability of ADIM during prone hip extension in patients with excessive lumbar lordosis. The muscle onset time during ADIM application revealed that only the GM of the LHLA group showed a significant difference (*P* <0.01), whereas the other muscles in the LNLA group showed no significant difference. An inter-group comparison revealed that the GM muscle onset time showed a significant difference (*P* <0.01). This result may be explained by the fact that ADIM affected the lumbar lordotic angle of the LHLA group to the neutral position and normalized the alignment of the pelvis. This alignment had a positive effect on the GM muscle contraction onset time, which is the most important action during prone hip extension.

The muscle contraction onset sequence during hip extension is clinically important, and a change in the muscle contraction onset sequence could cause back pain or pelvic dysfunction. The ideal muscle contraction onset sequence during hip extension was reported to be the GM followed by HAM and then LES [17]. However, Sakamoto et al. [9] reported that the muscle contraction onset sequence was HAM followed finally by GM during prone hip extension in 31 healthy subjects. Similar to the previous study [9], our results showed that the muscle contraction onset sequence, regardless of ADIM application, was HAM followed by LES and then GM in the LNLA group. However, the muscle contraction onset sequence changed from LES, HAM, and GM to GM, HAM, and LES by the application of ADIM in the LHLA group. The reason for this seems to be that ADIM application to control the imbalance of the lumbar region and pelvis promoted the contraction of the abdominal muscle, which prevented forward movement of the spine and improved the stability of the lumbar region. This positively affected the LES muscle activity and caused a change in the muscle contraction onset sequence.

A previous study of the clinical significance of the muscle activity and onset time of GM reported a delay in onset time of GM while patients with sacroiliac joint pain were supported on one leg [18]. The sacroiliac joint plays an important role in delivering the weight and ground force between the lower limbs and trunk. Vilensky et al. [19] suggested that the normal proprioceptive input of the mechanoreceptor in the sacroiliac joint is critical for the maintenance of standing posture. The delay in GM muscle activity and muscle contraction onset time affects the stability of the sacroiliac joint during functional activities, which causes lower back and sacroiliac pain [20, 21]. To address this problem, ADIM was applied during hip extension, which contributed to the internal stabilization of the lumbar region and promoted GM muscle activity. Furthermore, a previous study reported that ADIM application increased the core stability by contracting the transverse abdominalis and the lumbar multifidus, which effectively improved lumbar stability [21]. Shafik et al. [21] claimed that the increasing intra-abdominal pressure from abdominal muscle contraction could increase GM muscle activity. Similarly, the present study showed that ADIM application in the LHLA group decreased the lumbar lordotic angle. Furthermore, we observed that using ADIM with the pressure biofeedback unit had a positive effect on GM muscle contraction onset time. Therefore, using ADIM with pressure biofeedback during the hip extension exercise is effective for preventing an excessive lumbar lordotic angle, and may also reduce HAM and LES activation.

This study had several limitations. First, we included subjects with hyperlordosis but no lower back pain, thus the results may not be applicable to patients with pain or dysfunction in the lumbar and pelvic regions. Second, although we used a metronome to steadily control the hip extension speed, the speed of hip extension was not controlled for every subject. Third, the muscle activities of the abdominal muscles, diaphragm, and pelvic floor muscles were not measured during ADIM, and so the influence of these muscles could not be quantified. Therefore, further studies are needed to investigate the effects of ADIM application on the contraction onset time of the abdominal and pelvic floor muscles, as well as the trunk and hip extensors, during prone hip extension.

## Conclusions

This study aimed to investigate the changes in the muscle onset time of the HAM, GM, and LES after ADIM application during hip extension in 40 subjects (20 in the LHLA group and 20 in LNLA group), and to verify the effects of ADIM on lordosis to determine its clinical usefulness.

The results of this study are as follows: i) the difference in the muscle contraction onset time during ADIM application revealed that only the GM in the LHLA group showed a significant difference, and ii) the inter-group comparison demonstrated that only the GM muscle contraction onset time showed a significant difference. Therefore, the results of this study partially support the hypothesis that the ADIM application in lumbar lordosis would affect the onset time of GM during hip extension, resulting in a significant difference between the LHLA and LNLA groups. Based on the above findings, the application of ADIM during hip extension was more effective in improving GM muscle contraction onset time in subjects with lumbar lordosis.

## Abbreviations

ADIM: Abdominal drawing-in maneuver
ASIS: Anterior superior iliac spine
EMG: Electromyography
GM: Gluteus maximus
HAM: Hamstring
LES: Lumbar elector spinae
LHLA: Lumbar hyperlordotic angle
LNLA: Lumbar normal lordotic angle
PSIS: Posterior superior iliac spine
SD: Standard deviation.
